# Interocular Symmetry Analysis of Corneal Elevation Using the Fellow Eye as the Reference Surface and Machine Learning

**DOI:** 10.3390/healthcare9121738

**Published:** 2021-12-16

**Authors:** Shiva Mehravaran, Iman Dehzangi, Md Mahmudur Rahman

**Affiliations:** 1 Department of Biology, School of Computer, Mathematical and Natural Sciences, Morgan State University, Baltimore, MD 21251, USA; 2 Center for Computational and Integrative Biology, Department of Computer Science, Rutgers University, Camden, NJ 08102, USA; i.dehzangi@rutgers.edu; 3 Department of Computer Science, School of Computer, Mathematical and Natural Sciences, Morgan State University, Baltimore, MD 21251, USA; md.rahman@morgan.edu

**Keywords:** unsupervised machine learning, clustering, cornea, corneal topography, interocular symmetry, corneal elevation, keratoconus

## Abstract

Unilateral corneal indices and topography maps are routinely used in practice, however, although there is consensus that fellow-eye asymmetry can be clinically significant, symmetry studies are limited to local curvature and single-point thickness or elevation measures. To improve our current practices, there is a need to devise algorithms for generating symmetry colormaps, study and categorize their patterns, and develop reference ranges for new global discriminative indices for identifying abnormal corneas. In this work, we test the feasibility of using the fellow eye as the reference surface for studying elevation symmetry throughout the entire corneal surface using 9230 raw Pentacam files from a population-based cohort of 4613 middle-aged adults. The 140 × 140 matrix of anterior elevation data in these files were handled with Python to subtract matrices, create color-coded maps, and engineer features for machine learning. The most common pattern was a monochrome circle (“flat”) denoting excellent mirror symmetry. Other discernible patterns were named “tilt”, “cone”, and “four-leaf”. Clustering was done with different combinations of features and various algorithms using Waikato Environment for Knowledge Analysis (WEKA). Our proposed approach can identify cases that may appear normal in each eye individually but need further testing. This work will be enhanced by including data of posterior elevation, thickness, and common diagnostic indices.

## 1. Introduction

The cornea is the dome-shaped layer of transparent tissue at the frontmost part of the eye globe, and its main function is to provide 75% to 80% of the refractive power of the eye [1,2,3]. In the frontal view, the cornea is almost circular in outline with a horizontal diameter of about 11.0–12.0 mm horizontally and 10.0–11.0 mm vertically [3]. Given the pivotal role of the cornea in vision, even small deviations from normal and subtle imperfections in the transparency and shape of the cornea can disturb the quality of the retinal image. Therefore, accurate measurement of various corneal properties such as its curvature, thickness, and elevation is an integral part of a comprehensive eye exam.

Early attempts at describing the corneal shape date back to 1619 when Scheiner used glass balls of known diameters to measure the curvature of the cornea [4]. Until quite recently, the description of the corneal shape was limited to local metrics of the corneal curvature as measured with manual keratometers and single-point measurements of the corneal thickness with ultrasound pachymeters. Technological advances in ophthalmology have provided us modern systems that perform computer analysis of photographs taken from the entire surface of the cornea, and convert the data to color-coded contour maps [5]. Today, corneal topographic categories such as “round”, “oval”, “bow-tie”, and “irregular”, that were originally described by Bogan et al. [6] in 1990 and further expanded by Rabinowitz et al. [7] in 1996 are well known to practitioners. Since their introduction, computerized imaging systems have greatly enhanced our understanding of the corneal topography in normal and disease conditions. However, identifying corneal degenerative changes in early subclinical stages remains a challenge [8,9], and there is an active area of research to develop discriminative algorithms and finetune diagnostic criteria using state-of-the-art corneal imaging systems.

The Pentacam (Oculus GmBH, Wetzlar, Germany) is a popular projection-based anterior segment imaging device that utilizes a high-resolution Scheimpflug camera that scans the anterior segment by rotating 360° around the center [10]. The system captures data from 25,000 distinct elevation points within 2 s which are used to generate a 3-dimensional virtual model of the anterior segment. Once image processing is complete, the user can choose to review various maps and displays such as the sagittal curvature, pachymetry, and elevation maps of the anterior and posterior cornea, which compose the default 4-map display. Originally, the elevation of each point on the corneal surface is measured as its distance from a reference plane tangent to the corneal apex. This is quite similar to terrain topography, where elevation is defined as the distance above sea level. However, to make subtle surface variations discernible, the displayed data is a recalculation of the raw data to express the perpendicular distance from a sphere of variable diameter and position that best fits each individual cornea.

Currently available diagnostic algorithms and classification systems are mainly based on unilateral data [8,11,12,13]. Since there is wide variation in the normal population that define their reference ranges, they have shown suboptimal performance in discriminating normal corneas from subclinical forms of disorders [14]. This is while measures of normal fellow corneas are strongly correlated [15,16,17], contralateral eyes are highly symmetric [18,19,20,21,22], and there is consensus that lack of symmetry should be interpreted as a red flag warranting reevaluation or further testing [20,23,24,25,26,27]. Nonetheless, our understanding of corneal symmetry is limited to single-point metrics (e.g., elevation at the apex, corneal thickness at the thinnest point) and local indices (e.g., simulated keratometry in the steep and flat axes); the color-coded patterns have not been classified or described, and no multi-feature or global indices have been developed yet. Some other limitations of extant literature are that studies were mostly clinic-based with small sample sizes of defined groups that are not representative of the general population, and they used relative measures of elevation displayed by the system rather than the actual elevation (height) data.

This study was designed as a proof-of-concept study for using fellow eyes as the reference surface using raw Pentacam elevation data from a large population-based sample (including normal and abnormal) with two main goals: (1) describe pancorneal symmetry patterns observed in difference colormaps, and (2) cluster the data by applying machine learning techniques. Proving the feasibility of this approach is the first step in creating a novel diagnostic index for identifying cases with subtle changes and to assess longitudinal changes in the same eye.

## 2. Materials and Methods

The proposal of this secondary data analysis study was reviewed and approved by the Institutional Review Board of Morgan State University. The deidentified data was obtained from the Shahroud Eye Cohort Study (ShECS) which is an observational cohort of adults between the ages of 40 and 64 years at first enrollment [28,29]. To date, three phases of the study have been completed at 5-year intervals. Of the 6311 Shahroud residents who were invited to the study in 2009, 5190 participated (82.2% participation rate), completed the interview, and had a comprehensive eye examination including anterior segment imaging with the Pentacam. For this study, we used baseline data including the deidentified cohort database (containing demographic variables including age and gender) and Pentacam elevation files directly exported from the device. The only inclusion criterion was having both the right and left eye elevation files (bilateral cases). 

Data management was done in the Anaconda3 platform using various packages of Python version 3.7.4 in the Jupyter Notebook (server version 6.0.1). Waikato Environment for Knowledge Analysis (WEKA) version 3.8.4 was used for unsupervised machine learning and cluster analyses of the data and engineered features [30].

### 2.1. Creating Pancorneal Difference Matrices

**Figure 1:**
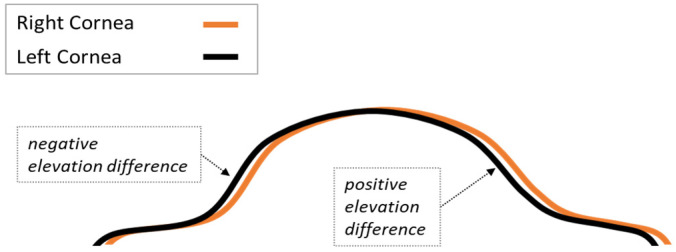
Schematic presentation of using the contralateral cornea as the reference surface for measuring elevation and assessing elevation symmetry between fellow eyes. Highly symmetric corneas should fit each other, and hypothetically, there will be zero distance between them. The higher the asymmetry, the bigger the area between the two surfaces.

For this step, first the IDs of right and left eyes were matched using Python’s *fnmatch* function to identify cases with bilateral data. Then the 141 × 141 matrix of anterior elevation values were extracted from each Pentacam elevation file. Each data point in the 141 × 141 anterior elevation matrix corresponds to an area of 0.1 × 0.1 mm; therefore, each matrix provides a coverage of 14 × 14 mm centered on the corneal apex (x = 0, y = 0 coordinates). The process for creating the fellow-eye difference matrices were relatively similar to what has been described by Cavas-Martínez et al. [22] who assessed shape symmetry in a sample of 33 normal cases. For each matched pair, the left eye matrix was rotated 180° around its Y axis using the NumPy *flip* function to account for the mirror symmetry between fellow eyes. Then, the right eye matrix was subtracted from the flipped left eye matrix. Figure 1 provides a schematic illustration of how the contralateral eye becomes the reference surface when raw elevation data are subtracted to create a fellow-eye difference matrix.

### 2.2. Creating Elevation Difference Colormaps

The difference matrices created in the previous step were color-coded to 2-dimensional colormaps. Using the Matplotlib and Seaborn packages, we assigned the spectral color palette because it resembles the ones routinely used in corneal topography. As such, the scale range was set from extreme negative (plotted in dark red) to extreme positive (plotted in dark blue) and the center 0 point was plotted as bright yellow. Therefore, deviation from the middle of the scale to either side could be illustrated with ascending darker colors.

### 2.3. Feature Engineering

To exclude extreme outliers in the corneal periphery that could be due to the effect of the limbus, eyelids, nose shadow, pterygium, and/or data extrapolation, elevation difference matrices were masked to only keep the data in the central 6.0 mm zone of the cornea (2821 data points per case). This zone was further divided to four smaller concentric zones with diameter sizes of 2.0, 3.0, 4.0, and 5.0 mm. The data within these five zones (2-dimensional arrays) were then flattened to a single dimension using the *flatten* function of NumPy and compiled into a single data frame in which there was one row of data per participant, and the columns represented the coordinates of the 2-dimensional masked matrix. The data in each row were summarized into their descriptive statistics including skew, absolute skew, kurtosis, mean, standard deviation of the mean, absolute mean (average of absolute means), median, absolute median, minimum, maximum, absolute maximum (the larger of maximum and absolute minimum), range, and central 95% range. The sums of negative and positive elevation difference values (Figure 1) were used to calculate the negative and positive volumes, respectively, as well as the sum of the two volumes (Total Volume) and the absolute difference between the two volumes (Volume Difference) as a measure of intraindividual asymmetry.

### 2.4. Cluster Analysis

In the next step, the data from difference matrices and their descriptive statistics were used as features for unsupervised machine learning analysis in WEKA. Different combinations were tested with different clustering algorithms such as the simple k-means and the simple expectation-maximization (EM) algorithms, and in some iterations, principal component analysis (PCA) was applied first for feature reduction. The outputs were inspected and compared in terms of the distribution of cases within each cluster, number of clusters, and the summary statistics of difference features.

### 2.5. Adding Other Indices

To make comparisons with the literature, we extracted the apical and minimum corneal thickness, maximum (simulated keratometry at the steep meridian) and mean (average of the keratometry in the steep and flat meridians) keratometry readings, and corneal astigmatism and computed the absolute interocular difference for these continuous variables. Pentacam also generates two categorical parameters, namely the quality specification (QS) and the keratoconus score (KKS) for each examined eye, which indicate the quality of the data and normality of the cornea, respectively. To examine the agreement between our clustering results and Pentacam-assigned categories, these parameters were extracted, recoded, and combined to create four bilateral categories with QS indicated as OK (Tables S1 and S2).

## 3. Results

A total of 9303 Pentacam elevation files were available; 4670 right eyes and 4633 left eyes. Matching the right and left data files by their study ID resulted in 4615 bilateral cases, two of which were excluded due to insufficient data points (computations returned NULL), and 4613 were included in the analysis. The mean age of this sample was 50.9 ± 6.3 years, and 41% were male.

### 3.1. Symmetry Patterns in Colormaps

**Figure 2:**
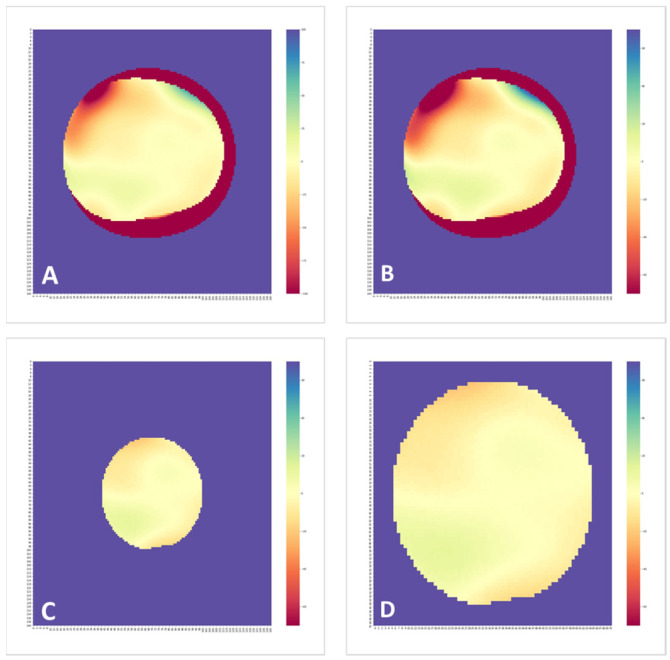
Fellow-eye elevation difference colormaps of a 53-year-old woman. The full 140 × 140 difference matrix using a ±100 µm scale (**A**) and a ±70 µm scale (**B**) show sharp contours in the periphery which were eliminated by masking the central 6.0 mm (**C**) and cropping out the extra data (**D**).

Figure 2 illustrates four different interocular elevation difference colormaps of the same individual; the peripheral outliers were removed by masking the difference matrices to the central 6.0 mm zone, and the overall visualization was improved by setting the scale to ±70 µm and cropping the image.

**Figure 3:**
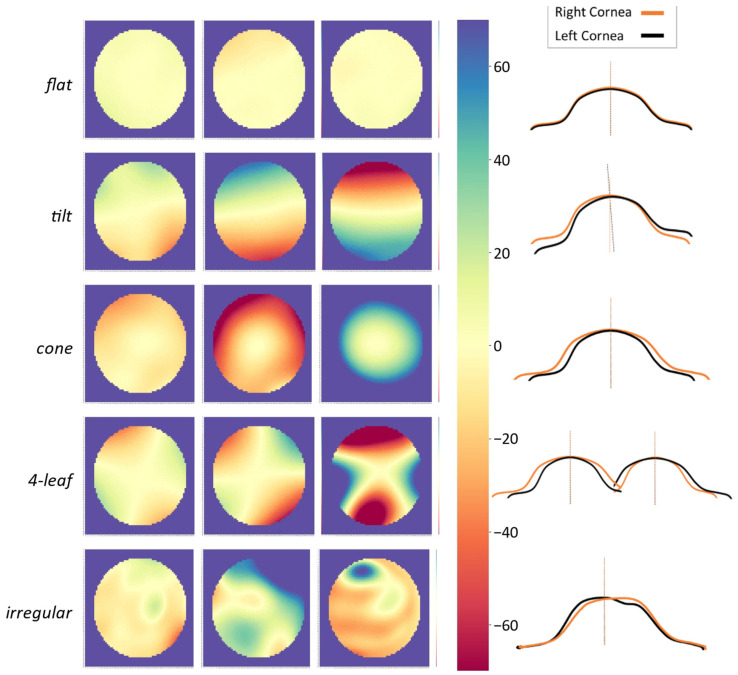
Sample 6.0 mm colormaps of common patterns observed in the elevation difference colormaps. The same ±70 µm scale (shown in the middle) was applied to all colormaps. The schematics on the right demonstrate how the fellow corneas fit in each category. In the flat pattern, the fellow corneas fit well, and there is minimum distance between them. In the tilt pattern, half of one cornea is below and the other half is above its fellow cornea. In the cone pattern, one cornea is steeper that its fellow cornea, and the area between the two surfaces increases from the center to the periphery. In the 4-leaf pattern, one cornea is steeper in a given meridian and flatter in the perpendicular meridian compared to its fellow cornea.

In reviewing the color-maps generated from fellow-eye difference data, we found a monochrome yellow circle to be the most common pattern showing that the interocular difference is zero or very close to zero, and the fellow corneas fit nicely with very little or no gap between them; this was named “flat” (Figure 3). Other commonly discernible patterns of interocular difference colormaps were named “tilt”, “cone”, “4-leaf”, and “irregular”. As illustrated in Figure 3, the pattern we named “tilt” demonstrated a semicircle of negative values on one side and a semicircle of positive values on the other side, separated by a yellow band (zero or near zero values). This pattern could be indicative of a difference in the imaging or visual axis between fellow eyes, and one eye is off-axis. The “cone” pattern would appear in cases where one cornea is steeper than the other, and the gap between them increases from the center to the periphery; this is the pattern one would expect to see in central keratoconus. The “4-leaf” pattern can be attributed to situations where the cornea in one eye is steeper in a certain meridian and flatter in the perpendicular meridian; these could be cases of direct symmetry especially in the presence of corneal astigmatism. Symmetry patterns that did not fit any of these categories were assigned to the “irregular” group.

### 3.2. Data Exploration and Feature Engineering

**Figure 4:**
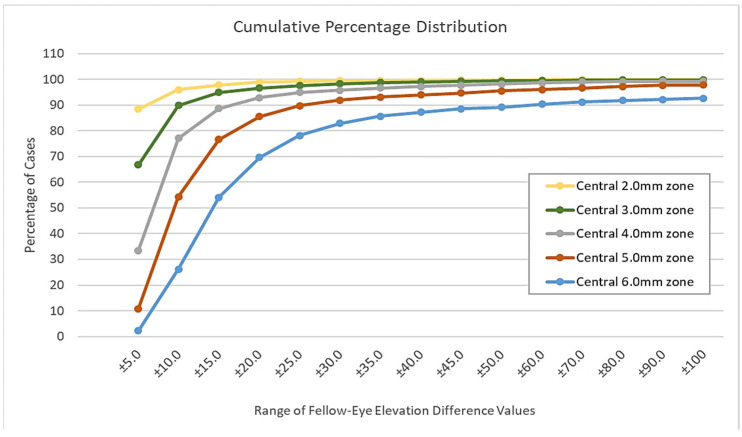
Cumulative percentage of cases that had all data points within a given range.

Figure 4 illustrates the cumulative percentage of cases in which the minimum and maximum interocular elevation difference (i.e., values of all data points) in each of the five studied zones was within the specified range. For example, all data points in the central 2.0 mm zone were within ±5.0 µm in 88.4% of the cases, within ±10.0 µm in 96.0%, and within ±15.0 µm in 97.6% of cases. In case of the central 3.0 mm zone, all data were within ±10.0 µm in 90.0% and within ±25.0 µm in % 97.6% of cases. In case of the central 6.0 mm, all data were within ±60.0 µm in 90.0%, and within ±100.0 µm in 92.7% of cases.

In the total sample of 4613 cases, mean elevation difference at the (0, 0) coordinates was 0.04 ± 2.0 µm (central 95% range: 7.8 µm). The descriptive statistics (measures of central tendency and variability) are summarized as their mean, standard deviation of the mean, and the central 95% range in Table S3. Both the mean and variance of the data increased in larger, more peripheral zones.

### 3.3. Unsupervised Machine Learning

**Figure 5:**
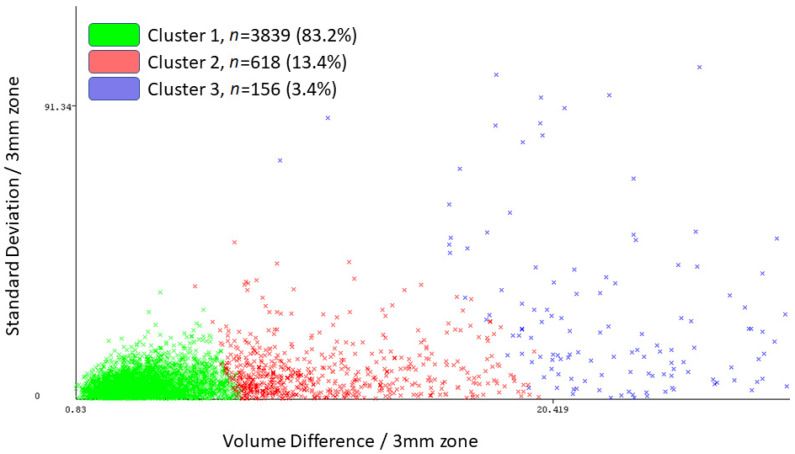
WEKA visualization output using simple k-means clustering. Attributes used in this model included the central 95% range of the 6.0 mm zone and the mean, standard deviation of the mean, and volume difference of the central 3.0 mm zone.

Table 1 and Figure 5 present the results of simple k-means clustering in WEKA with the following attributes: the central 95% range in the 6.0 mm zone and the absolute mean, standard deviation of the mean, and volume difference in the central 3.0 mm zone. The full sample was grouped into three clusters with 3839 (83.2%) in Cluster 1, 618 (13.4%) in Cluster 2, and 156 (3.4%) in Cluster 3; mean elevation difference at the (0, 0) coordinates was −0.0005 ± 0.32 µm, −0.016 ± 0.29 µm, and 1.12 ± 10.76 µm in Cluster 1, 2, and 3, respectively.

**Figure 6:**
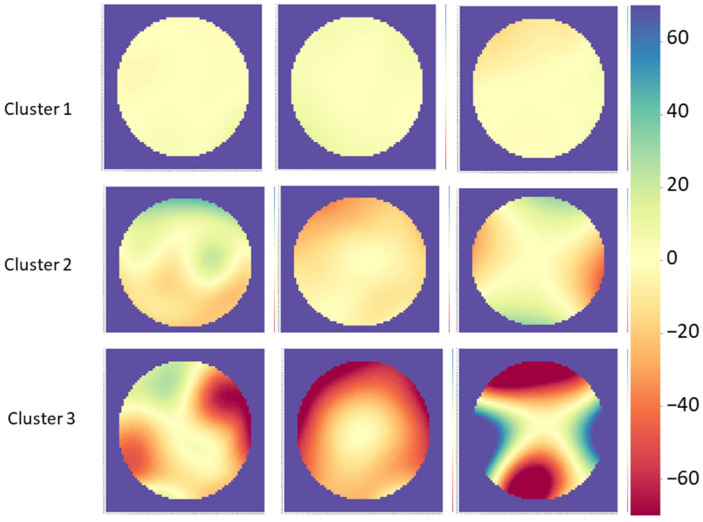
Sample 6.0 mm colormaps of three random cases from each of the three clusters. The scale in all colormaps is the ±70 µm scale shown on the right. These three clusters were created in using the simple k-means clusterer in WEKA and the following attributes: central 95% range of the 6.0 mm zone and the absolute mean, standard deviation of the mean, and volume difference of the central 3.0 mm zone. Cluster 1 corresponds with normal corneas and all maps showed the flat pattern. Other patterns were observed in cluster 2 and 3, although the degree of asymmetry was greater in the latter group.

Figure 6 illustrates the central 6.0 mm interocular elevation difference maps of three random samples from each of the three clusters. In Cluster 1, the colormap pattern was “flat” in all cases; the other patterns appeared in Cluster 2 with lighter colors and in Cluster 3 with darker colors.

Table 2 summarizes the summary statistics of the interocular anterior elevation differences in the 3 clusters within the five studied central corneal zones. Overall, both the mean and the spread of the values were smallest in Cluster 1 and highest in Cluster 3. They were also higher in larger, more peripheral corneal zones within each cluster.

### 3.4. Assessing Clusters Using Parameters Other Than Elevation

Table 3 presents summary statistics of the studied corneal thickness and curvature parameters in the right eyes, left eyes, and the absolute interocular difference in the total sample (*n* = 4613) and the 3 clusters. Similar to elevation data, both the mean and spread were smallest in Cluster 1 and highest in Cluster 3.

From the total sample of 4613, 571 cases had imaging errors (QS of 1 or 2, see Table S1) in at least one eye, and they were excluded from the comparison with Pentacam normality indices. As indicated in the top section of Table 4, of the 2975 cases in the Bilateral-normal/QS-OK category (64.5% of the total sample), 2696 (90.6%) were in Cluster 1 (the cluster with the least interocular differences). However, from this same category, 22 (0.7%) were in Cluster 3 (the cluster with the highest levels of difference between fellow eyes). Central 6.0 mm fellow-eye elevation difference maps of these cases are illustrated in Table 5 and Table 6. All 10 cases illustrated in Table 5 have 1.0 D or more interocular difference in corneal astigmatism; in 8 cases, the difference is 2.5 D or more. Cases #4 and #5 in Row 1 as well as case #4 in Row 2 also show considerable interocular differences in terms of corneal thickness at the apex and thinnest point. Among the remaining 12 cases (Table 6), all three cases in Row 3 have 2.5 D or more interocular difference in maximum keratometry, and case #1 in Row 3 shows more than 30 µm corneal thickness difference between fellow eyes. The four cases shown in Row 4 have 17 µm or more interocular thickness difference either at the apex, the thinnest point, or both. Finally, the five cases in Row 5 have 13.0 µm or more absolute maximum elevation difference.

## 4. Discussion

One of the main objectives of this study was to create fellow eye anterior elevation difference colormaps and suggest descriptive names for discernible patterns. As expected, the most common pattern was “flat” showing that the interocular difference is zero or very close to zero and the fellow corneas fit nicely with very little or no gap between them (Figure 3). The “tilt” pattern could be attributed to a difference in the imaging or visual axis between fellow eyes; identifying this pattern could have implications in evaluating strabismus, prescribing corrective eyeglasses, or, as suggested by Fathy et al. [31], in screening for keratoconus. The “cone” pattern is expected in keratoconus, especially central forms. The “4-leaf” pattern can be attributed to cases of direct symmetry especially in the presence of corneal astigmatism; for these cases, creating fellow-eye difference matrices without flipping the left eye matrix could return one of the other patterns. Although the patterns of unilateral corneal topography maps have been studied and have accepted nomenclature [6,7], to the best of our knowledge, this is the first study to examine fellow-eye difference maps and give them descriptive names. Adding fellow-eye difference displays to corneal imaging systems can facilitate interocular symmetry review for eye care providers, and once they become familiar with the patterns and complete the learning curve, the approach has the potential to become an integral part of a comprehensive eye exam, especially for preoperative screening.

Recent studies of fellow-eye symmetry have looked at different corneal features and parameters including corneal biometrics [27,32], higher order aberrations [33], and corneal surface area [34]. A summary of the few studies that have examined anterior elevation symmetry is presented in Table 7 [21,25,35,36,37,38,39]. These studies greatly vary by methodology such as sample selection and size, the corneal topographer used for imaging, the reference surface used for measuring elevation, and the choice of elevation measure.

As summarized in Table 7, Falavarjani et al. [25] reported a mean interocular difference of 2.2 µm (range: 0 to 21.0 µm) and suggested that a difference greater than 17.4 µm (95th percentile) should be interpreted as a potential red flag. Their results can be compared to our 4.0 mm data summarized in Table 2. The mean absolute maximum (the larger of maximum and absolute minimum) was 6.8 ± 6.8 µm, 19.2 ± 16.0 µm, and 60.7 ± 52.1 µm in Clusters 1, 2, and 3, respectively, and 10.2 ± 16.5 µm in the total sample. Therefore, the average of 2.2 µm reported in their study is even smaller than what was observed for Cluster 1 (6.8 µm) which is the group with highest symmetry in our study. This is mainly due to methodological differences; they only included healthy eyes and calculated the interocular difference at only one single point (the maximum anterior) which was measured from a spherical reference surface that may have been different between fellow eyes.

The study by Durr et al. [21] was similar to ours in that they examined a large population-based sample. Methodological differences included using Orbscan IIz, applying exclusion criteria (no history of ocular disease, ocular surgery, or recent contact lens wear), and using a reference surface based on the average best fit sphere of all right and left eyes. The average anterior elevation difference in the 6.0 mm zone in their study ranged within ±6.0 µm. Because of the methodological differences mentioned above, as well as age differences between the samples of the two studies, their result is much smaller the mean range of 27.6 µm observed for the 6.0 mm zone in Cluster 1 of our study (Table 2).

The other five reports summarized in Table 7 were clinic-based comparative studies, that enrolled two or more sample groups, one being a normal control group and one a group of keratoconus patients. Saad et al. [35] used the Orbscan IIz with the reference surface set on the default float mode. Although the intergroup differences were statistically significant (both *p* < 0.001), the mean interocular differences they observed in the maximum anterior elevation and the anterior elevation at the thinnest point of the cornea (Table 7) were very close to zero in both groups. Galletti et al. [36] and Eppig et al. [39] used the Pentacam; the reference surface is not mentioned, but perhaps the default setting was used [40]. Galletti et al. [36] included the absolute interocular difference of the anterior elevation at the thinnest point. The central 90% range for this variable was 4.0 µm in the normal comparison group and 31.0 µm in the group that was labelled as keratoconus based on Pentacam diagnostic indices. Eppig et al. [39] examined another relative measure of anterior elevation which looks at the difference in elevation values between measurements made with a standard best fit sphere and an “enhanced” best fit sphere which is calculated from the 9.0 mm central data minus the 4.0 mm around the thinnest point [41]. Naderan at al. [37] appear to have set the device to use an 8.0 mm best fit sphere. The anterior elevation measure they examined in their study is described as the “maximum at the thinnest point” of the cornea “based on the data from the 3.0 mm annular corneal diameter ring”. They found a mean absolute interocular difference of 1.3 ± 0.7 µm (0.0 –7.0 µm) in the normal comparison group, 5.5 ± 4.8 µm (1.0–14.0 µm) in the keratoconus suspect group, and 14.0 ± 10.4 µm (1.0–36.0 µm) in the keratoconus group. The interocular differences observed by Henriquez et al. [37] were very similar to that reported by Naderan et al. [37]. In both cases, the interocular differences are far smaller than what was observed in our study, which again, similar to the study by Falavarjani et al. [25], could be attributed to methodological differences and the use of a variable reference surface.

This study (bottom row in Table 7) is novel in multiple ways. Firstly, the fellow cornea was used as the reference surface (Figure 1). This was based on the hypothesis that doing so would allow one to discern subtle interocular differences that may not be obvious when comparing two separate elevation maps, especially if their measurements are based on different reference surfaces. Secondly, the symmetry data was pancorneal and not limited to one or two points. The number of corresponding data points in the central 2.0 mm, 3.0 mm, 4.0 mm, and 5.0 mm of the cornea were 317, 709, 1257, and 1961, respectively, and the central 6.0 mm was represented by 2821 data points. Thirdly, from the subtraction matrix of each individual, multiple features representing their central tendency and variability (skew, mean, central 95% range, total volume, etc.) in the 2.0–6.0 mm zones of the cornea were engineered and used as attributes in machine learning and clustering algorithms. Another strength of this study is its large population-based sample (*n* = 4613) and inclusion of all cases.

Different combinations of a multitude of features were tested in different iterations with WEKA. To maintain simplicity and allow comparison with other studies, the next steps of the analyses were done with a 3-cluster output. As demonstrated in Table 2, both the mean and the standard deviation (spread) of the summary statistics were significantly different between the three groups; values were lowest in Cluster 1 (best symmetry) and highest in Cluster 3 (least symmetry). A similar trend was observed when the three clusters were compared in terms of corneal thickness and curvature indices (Table 3). This is because corneal features are strongly correlated. In fact, elevation-based topographers, such as the Pentacam, capture elevation data directly, while anterior and posterior corneal power data are computed from the elevation data of their corresponding surface and corneal thickness is the elevation distance between the two corneal surfaces. 

A summary of interocular symmetry studies examining measures of corneal thickness and curvature is presented in Table 8 [23,24,35,37,38,39,42,43,44]. Comparison of the values shows that Cluster 1 corresponds with normal groups. As such, mean interocular differences in central and minimum corneal thickness were 8.0 µm and 8.1 µm in Cluster 1, respectively, and they ranged between 4.3 µm and 11.0 µm in the normal groups of other studies. In terms of maximum, minimum, and mean keratometry, all three values were around 0.3 D in Cluster 1, and the range reported for the normal groups summarized in Table 8 is between 0.2 D and 0.4 D. However, as demonstrated in Table 4, 11.7% of Cluster 1 cases were red-flagged by Pentacam. Since their colormap patterns were “flat”, this mismatch is probably due to the fact that only anterior corneal elevation data were used for clustering, and therefore, abnormalities in the corneal thickness and posterior corneal surface were overlooked. A similar comparison shows that Cluster 2 is comparable to the keratoconus suspect group in the study by Naderan et al. [37]; other studies did not have an intermediate or suspect group. In Cluster 3, mean interocular differences were 41.1 µm and 58.3 µm for central and minimum corneal thickness, respectively, while the values in keratoconus groups of other studies are in the range of 25.9–34.0 µm and 30.2–39.8 µm for central and minimum corneal thickness, respectively. The interocular differences in maximum and mean keratometry readings are lower in Cluster 3 compared to the keratoconus groups in other studies, and minimum keratometry is in the mid-range. Also, contrary to all other groups, the difference in minimum keratometry is higher than that of maximum keratometry. The lack of agreement between Cluster 3 and other groups is probably because the sample in our study was the general population and mirror symmetry was assumed. As such, there may be highly asymmetric cases (albeit clinically normal) due to other reasons such as anisoastigmatism, anisorule, and/or direct symmetry patterns [22,45].

Anisoastigmatism is defined as an interocular difference of 1.0 D or more in refractive astigmatism [46,47,48]. The interocular difference in anterior corneal astigmatism was 3.4 D in Cluster 3, but the range in the keratoconus groups of other studies is only 1.8–2.1 D (Table 8). Twenty-two cases in Cluster 3 were found to be bilaterally normal by Pentacam’s built-in algorithms (Table 4), and 10 of them had anisoastigmatism (Table 5). The common discernable pattern in this group (Table 5 and Table 6) was “tilt” (Figure 1) which can be due to interocular differences in angle kappa or how the apex, line of sight, and measurement axis line up [40] or a displaced apex [49]. One way to examine this is the interocular difference in anterior elevation at (0, 0) coordinates; the mean of this index was −0.18 µm in this subsample of 22 cases, 0.04 in the total sample, and 0.0 in Cluster 1. To control for such an effect in future research, we will apply the iterative closest point transformation algorithm described by Fathy et al. [31] before subtracting data on corresponding points.

As mentioned earlier, this study had certain limitations that need to be addressed in the follow-up work. Firstly, despite the large sample size, the age range was limited to 40–64 years who might have higher levels of corneal irregularity than younger individuals [50]. In our future work, we will use data from a younger population-based cohort [51] and/or the general sample from a clinical database. Secondly, since this was a preliminary proof of concept study, clustering algorithms were provided with anterior elevation data only; this can explain the false negative and false positive cases described above. Also, to allow for simplicity and comparability, the number of clusters was limited to three. In future work, adding features derived from posterior elevation and thickness symmetry and previously developed diagnostic indices along with a larger (or automated) number of clusters could help improve the accuracy of the algorithm and facilitate classifying symmetry patterns.

## 5. Patents

The concept behind this work is under patent protection by Morgan State University.

## Figures and Tables

**Table 1:** WEKA output using simple k-means clustering. Attributes used in this model included the central 95% range of the 6.0 mm zone and the mean, standard deviation of the mean, and volume difference of the central 3.0 mm zone.

Attribute	Full *n* = 4613	Cluster 1 *n* = 3839	Cluster 2 *n* = 618	Cluster 3 *n* = 156
Central 95% Range/6.0 mm	20.7	14.1	42.1	97.0
Absolute Mean/3.0 mm	0.8	0.6	1.2	4.5
Standard Deviation/3.0 mm	2.0	1.3	3.7	11.4
Volume Difference/3.0 mm	5.7	4.3	8.2	30.8

Clustering Model: k-means; Number of iterations: 30; Within cluster sum of squared errors: 34.2.

**Table 2:** Mean and standard deviation of summary statistics of anterior elevation difference (in µm) between corresponding points on fellow eyes in the total sample and the three clusters within the five concentric central corneal zones.

Zone	Statistic	Full Sample *n* = 4613	Cluster 1 *n* = 3839	Cluster 2 *n* = 618	Cluster 3 *n* = 156
2.0 mm	Abs-Mean	0.5 ± 2.1	0.3 ± 0.3	0.6 ± 0.6	3.5 ± 10.7
	SD	1.3 ± 2.0	0.9 ± 0.4	2.2 ± 1.2	7.4 ± 8.1
	Min	−2.8 ± 3.6	−2.0 ± 1.1	−4.8 ± 2.6	−15.4 ± 11.4
	Max	2.9 ± 6.1	2.0 ± 1.2	5.0 ± 3.2	17.2 ± 28.3
	Range	5.7 ± 8.6	3.9 ± 1.6	9.8 ± 4.7	32.5 ± 34.3
	Abs-Max	3.7 ± 6.5	2.5 ± 1.1	6.2 ± 3.0	21.9 ± 28.1
3.0 mm	Abs-Mean	0.8 ± 2.3	0.6 ± 0.5	1.2 ± 1.1	5.5 ± 11.2
	SD	2.0 ± 3.1	1.3 ± 0.5	3.7 ± 1.5	12.5 ± 12.0
	Min	−4.8 ± 6.6	−3.2 ± 1.8	−8.7 ± 4.2	−28.0 ± 21.9
	Max	2.9 ± 6.1	2.0 ± 1.2	5.0 ± 3.2	17.2 ± 28.3
	Range	9.6 ± 14.3	6.4 ± 2.5	17.5 ± 6.7	57.6 ± 54.0
	Abs-Max	6.3 ± 9.8	4.2 ± 1.8	11.1 ± 4.6	38.5 ± 38.3
4.0 mm	Abs-Mean	1.3 ± 2.8	0.9 ± 0.7	1.8 ± 1.6	8.0 ± 12.6
	SD	2.9 ± 4.5	1.9 ± 0.9	5.6 ± 2.2	18.9 ± 16.3
	Min	−7.5 ± 10.5	−5.0 ± 2.9	−14.2 ± 7.0	−44.0 ± 34.2
	Max	7.8 ± 15.4	5.0 ± 6.9	15.1 ± 16.5	46.7 ± 53.4
	Range	15.3 ± 23.0	10.0 ± 7.4	29.3 ± 18.3	90.7 ± 76.1
	Abs-Max	10.2 ± 16.5	6.8 ± 6.8	19.2 ± 16.0	60.7 ± 52.1
5.0 mm	Abs-Mean	1.8 ± 3.5	1.3 ± 1.1	2.6 ± 2.5	11.0 ± 14.4
	SD	4.4 ± 6.9	2.8 ± 2.7	8.6 ± 6.0	26.9 ± 21.0
	Min	−11.1 ± 15.1	−7.3 ± 4.3	−21.7 ± 11.3	−63.3 ± 48.0
	Max	13.6 ± 35.5	9.1 ± 27.1	26.8 ± 46.7	71.8 ± 78.3
	Range	24.7 ± 43.3	16.4 ± 27.8	48.5 ± 48.4	135.1 ± 105
	Abs-Max	17.4 ± 36.4	11.7 ± 26.8	33.8 ± 45.7	92.1 ± 75.3
6.0 mm	Abs-Mean	2.6 ± 4.7	1.9 ± 2.3	4.0 ± 5.0	14.0 ± 16.9
	SD	7.3 ± 13.3	5.0 ± 9.6	13.8 ± 15.2	38.0 ± 28.2
	Min	−16.0 ± 20.9	−10.7 ± 6.4	−31.5 ± 18.1	−85.4 ± 64.6
	Max	31.3 ± 92.6	23.8 ± 83.4	53.3 ± 109.0	129.6 ± 149.8
	Range	47.3 ± 99.3	34.5 ± 84.3	84.7 ± 110.6	214.9 ± 175.5
	Abs-Max	36.8 ± 92.4	27.6 ± 82.8	64.1 ± 106.7	154.4 ± 141.8

All *p* < 0.001; ANOVA comparing the mean in the three clusters. Abs-Mean: mean of absolute mean differences; SD: standard deviation; Min: minimum; Max: maximum; Abs-Max: the larger of maximum and absolute minimum.

**Table 3:** Mean ± standard deviation of the corneal thickness and curvature indices in the right end left eyes, and their absolute interocular difference in the total sample and the three clusters.

Parameter		Total	Cluster 1	Cluster 2	Cluster 3	*p*-Value *
		*n* = 4613	*n* = 3839	*n* = 618	*n* = 156	
Apical Thickness (µm)	OD	529.9 ± 33.7	530.9 ± 32.0	527.6 ± 34.5	515.4 ± 59.2	<0.001
	OS	530.6 ± 33.8	531.5 ± 32.2	527.9 ± 35.3	519.6 ± 55.4	<0.001
	i-dif	9.6 ± 13.2	8.0 ± 6.4	11.4 ± 11.1	39.7 ± 51.7	<0.001
Minimum Thickness (µm)	OD	524.5 ± 37.0	526.6 ± 32.1	521.2 ± 34.7	486.6 ± 93.7	<0.001
	OS	525.2 ± 35.6	527.2 ± 32.3	521.0 ± 35.9	493.5 ± 73.8	<0.001
	i-dif	10.3 ± 20.0	8.1 ± 6.4	12.0 ± 12.1	56.8 ± 89.5	<0.001
Maximum Keratometry (D)	OD	44.2 ± 1.7	44.1 ± 1.6	44.5 ± 1.9	45.5 ± 3.2	<0.001
	OS	44.2 ± 1.8	44.1 ± 1.6	44.6 ± 1.9	45.8 ± 3.8	<0.001
	i-dif	0.5 ± 0.8	0.3 ± 0.3	0.7 ± 0.6	2.4 ± 3.2	<0.001
Mean Keratometry (D)	OD	43.7 ± 1.7	43.7 ± 1.5	43.8 ± 1.8	44.0 ± 3.3	0.017
	OS	43.8 ± 1.7	43.7 ± 1.5	43.9 ± 1.8	44.3 ± 3.3	<0.001
	i-dif	0.4 ± 0.6	0.3 ± 0.2	0.6 ± 0.5	2.3 ± 2.5	<0.001
Corneal Astigmatism (D)	OD	0.9 ± 1.1	0.8 ± 0.5	1.3 ± 1.1	3.0 ± 4.2	<0.001
	OS	0.9 ± 1.1	0.8 ± 0.5	1.4 ± 1.3	3.0 ± 3.9	<0.001
	i-dif	0.5 ± 1.1	0.4 ± 0.3	1.0 ± 1.1	3.3 ± 4.7	<0.001

* ANOVA comparing the mean in the three clusters. D: diopter; OD: right eyes; OS: left eyes; i-diff: absolute difference between fellow eyes.

**Table 4:** Frequency distribution of the combined corneal abnormality categories in the full sample and the three clusters, and the mean (± standard deviation) interocular difference values of thickness and curvature measures.

Pentacam Category	Parameter	Total	Cluster 1	Cluster 2	Cluster 3
		(*n* = 4613)	(*n* = 3839)	(*n* = 618)	(*n* = 156)
Bilateral-normal QS-OK	*n*	2975 (64.5%)	2696 (90.6%)	257 (8.6%)	22 (0.7%)
	Ap-thick	8.2 ± 6.6	8.0 ± 6.2	10.0 ± 8.7	14.4 ± 10.3
	Min-thick	8.3 ± 6.5	8.1 ± 6.2	9.9 ± 8.6	12.5 ± 10.7
	MaxK	0.3 ± 0.3	0.3 ± 0.3	0.6 ± 0.6	0.9 ± 1.3
	MeanK	0.3 ± 0.3	0.3 ± 0.2	0.4 ± 0.4	1.4 ± 1.2
	Cor-ast	0.4 ± 0.5	0.3 ± 0.3	1.0 ± 1.0	1.9 ± 2.1
KS-abnormal QS-OK	*n*	684 (14.8%)	512 (74.9%)	144 (21.1%)	28 (4.1%)
	Ap-thick	9.5 ± 9.4	8.1 ± 6.7	11.4 ± 10.3	25.8 ± 22.1
	Min-thick	10.0 ± 11.8	8.0 ± 6.5	11.9 ± 9.7	36.3 ± 37.6
	MaxK	0.5 ± 1.0	0.4 ± 0.3	0.7 ± 0.7	2.3 ± 4.3
	MeanK	0.5 ± 0.7	0.3 ± 0.2	0.7 ± 0.6	2.0 ± 2.2
	Cor-ast	0.6 ± 1.3	0.4 ± 0.3	1.0 ± 1.1	3.3 ± 4.9
KCN-1-2 QS-OK	*n*	84 (1.8%)	30 (35.7%)	36 (42.9%)	18 (21.4%)
	Ap-thick	15.8 ± 14.1	8.6 ± 7.3	14.9 ± 10.5	29.6 ± 19.0
	Min-thick	16.9 ± 17.6	9.2 ± 7.2	14.4 ± 10.2	34.5 ± 27.6
	MaxK	1.2 ± 1.1	0.6 ± 0.4	1.1 ± 0.9	2.3 ± 1.6
	MeanK	0.9 ± 1.0	0.4 ± 0.3	0.9 ± 0.6	1.8 ± 1.6
	Cor-ast	1.0 ± 1.0	0.5 ± 0.4	1.0 ± 1.0	1.7 ± 1.5
KCN-3-4 QS-OK	*n*	10 (0.2%)	0 (0.0%)	4 (40.0%)	6 (60.0%)
	Ap-thick	33.2 ± 34.1		16.0 ± 6.8	44.7 ± 40.9
	Min-thick	29.4 ± 28.8		18.3 ± 9.8	36.8 ± 35.7
	MaxK	3.7 ± 4.4		2.1 ± 0.8	4.8 ± 5.6
	MeanK	3.2 ± 4.4		1.9 ± 0.5	4.0 ± 5.8
	Cor-ast	1.7 ± 1.3		1.9 ± 0.9	1.6 ± 1.6

Ap-thick: apical thickness (µm); Min-thick: minimum thickness (µm); MaxK: keratometry in the steep meridian (diopter); MeanK: average of keratometry in steep and flat meridians (diopter); Cor-ast: corneal astigmatism (diopter).

**Table 5:** Elevation difference colormaps of 10 Cluster 3 cases identified as bilaterally normal by Pentacam.

Parameter	1	2	3	4	5
Row 1	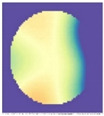	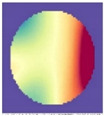	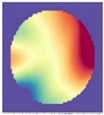	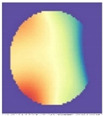	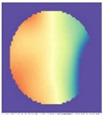
ΔAst	6.2	5.6	5.6	4.0	3.7
ΔmaxK	0.54	0.41	0.12	0.33	0.00
ΔpAx	7.0	3.0	19.0	23.0	34.0
ΔpThin	1.0	4.0	4.0	21.0	36.0
ΔmaxEle	12.0	17.0	23.0	13.0	18.0
Row 2	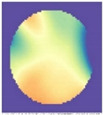	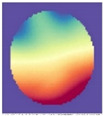	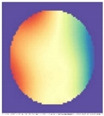	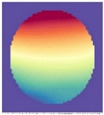	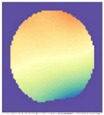
ΔAst	3.5	3.4	2.5	1.2	1.0
ΔmaxK	0.28	0.24	1.77	0.61	0.91
ΔpAx	6.0	1.0	5.0	25.0	11.0
ΔpThin	1.0	10.0	2.0	27.0	15.0
ΔmaxEle	12.0	12.0	7.0	27.0	14.0

Note: The scale in all colormaps is ±70 µm. Δ: interocular difference; Ast: anterior corneal astigmatism (diopter); maxK: maximum keratometry (diopter); pAx: pachymetry at the apex (µm); pThin: pachymetry at the thinnest point of the cornea (µm); maxEle: the larger of the maximum and absolute minimum elevation difference (µm).

**Table 6:** Colormaps of 12 cases in Cluster 3 that were identified as bilaterally normal by Pentacam.

Parameter	1	2	3	4	5
Row 3	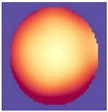	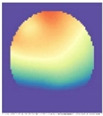	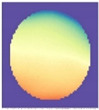		
ΔAst	0.5	0.7	0.6		
ΔmaxK	5.11	3.27	2.61		
ΔpAx	35.0	3.0	22.0		
ΔpThin	31.0	2.0	25.0		
ΔmaxEle	11.0	27.0	9.0		
Row 4	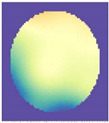	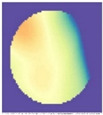	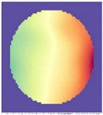	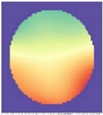	
ΔAst	0.2	0.1	0.1	0.9	
ΔmaxK	0.06	0.70	0.68	0.00	
ΔpAx	22.0	22.0	20.0	19.0	
ΔpThin	1.0	20.0	17.0	19.0	
ΔmaxEle	9.0	22.0	13.0	15.0	
Row 5	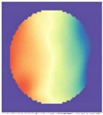	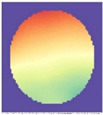	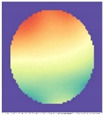	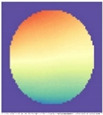	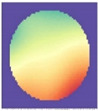
ΔAst	0.4	0.2	0.3	0.2	0.4
ΔmaxK	0.54	0.70	0.06	0.34	0.06
ΔpAx	9.0	0.0	10.0	14.0	7.0
ΔpThin	8.0	1.0	10.0	12.0	7.0
ΔmaxEle	23.0	17.0	17.0	16.0	13.0

Note: ±70 µm scale. Δ: interocular difference; Ast: anterior corneal astigmatism (diopters); maxK: keratometry in the steep meridian (diopters); pAx: pachymetry at the apex (µm); pThin: pachymetry at the thinnest point of the cornea (µm); maxEle: the larger of the maximum and absolute minimum elevation difference in the central 2.0 mm zone (µm).

**Table 7:** Summary of fellow-eye symmetry studies reporting measures of anterior corneal elevation.

First Author [Ref #]	Studied Sample	Reference Surface	Anterior Elevation Measure	Mean Interocular Difference (µm)
Falavarjani [25]	275 normal	Float BFS with auto diameter	Maximum in the central 4.0 mm	2.2 (range, 0–21)
Durr * [21]	3835 normal	Average BFS of all eyes	Average elevation in the central 6.0 mm ^†^	Range ± 6.0
Saad * [35]	51 normal 32 KCN	Default float BFS	Maximum/at thinnest point	Normal: 0.0 ± 0.0/0.0 ± 0.0 KCN: 0.02 ± 0.01/0.02 ± 0.01
Galletti [36]	177 normal 44 intermediate 121 KCN	No mention	At thinnest corneal location	Central 98% range Normal: 4.0 KCN: 31.0
Naderan [37]	306 normal 68 suspect 446 KCN	8.0 mm BFS	At thinnest point within the central 3.0 mm	Normal: 1.3 ± 0.7 KCS: 5.5 ± 4.8 KCN: 14.0 ± 10.4
Henriquez [38]	341 normal 50 high ammetropia 294 KCN	8.0 mm BFS	Maximum/at thinnest point	Normal: 1.4 ± 1.4/1.1 ± 1.0 KCN: 10.3 ± 11.0/8.7 ± 9.9
Eppig [39]	68 normal 350 KCN	No mention	Elevation deviation	Normal: 0.46 ± 0.39 KCN: 7.8 ± 7.4
Current Project	4615 general population	Raw data (the fellow eye)	Values and descriptive statistics of all corresponding points in the central 2.0–6.0 mm	See Table 2 and Table 3

* Used Orbscan IIz; all other studies used Pentacam; ^†^ Constructed from the average of each point in the whole sample. BFS: best fit sphere; KCN: keratoconus.

**Table 8:** Summary of fellow-eye symmetry studies reporting measures of corneal thickness and power.

First Author [Ref #]	Group	Corneal Thickness (μm)		Simulated Keratometry (D)			
		Central	Thinnest	Steep	Flat	Mean	Diff
Myrowitz * [23]	normal	-	8.0 ± 7.0	-	-	0.5 ± 0.4	-
Khachikian [24]	normal	8.8 ± 7.2	9.0 ± 8.3	-	-	-	-
Henriquez [42]	normal	10.2 ± 7.9	11.0 ± 8.2	0.3 ± 0.3	0.3 ± 0.2	-	-
	KCN	25.9 ± 24.1	30.2 ± 29.1	3.8 ± 4.2	2.7 ± 3.3	-	-
Henriquez [38]	normal	10.3 ± 7.9	11.0 ± 8.2	-	-	-	-
	KCN	25.9 ± 24.1	30.2 ± 29.1	-	-	-	-
Dienes [43]	normal	5.6 ± 4.9	6.6 ± 5.3	0.4 ± 0.4	0.4 ± 0.4	-	-
	KCN	30.1 ± 35.8	39.7 ± 36.4	4.4 ± 5.1	2.7 ± 3.6	-	-
Kovács [44]	normal	6.3 ± 6.9	6.9 ± 7.5	0.3 ± 0.2	0.3 ± 0.2	-	-
	KCN	29.9 ± 34.3	39.8 ± 29.1	3.3 ± 2.6	2.8 ± 3.1	-	-
Naderan [37]	normal	4.3 ± 1.6	5.9 ± 2.2	0.3 ± 0.2	0.2 ± 0.2	0.2 ± 0.2	0.1 ± 0.1
	suspect	12.8 ± 10.0	13.7 ± 10.9	1.0 ± 1.2	0.6 ± 0.8	0.7 ± 0.8	1.0 ± 0.8
	KCN	29.4 ± 28.5	33.6 ± 33.2	4.3 ± 4.2	3.4 ± 3.7	3.7 ± 3.8	1.8 ± 1.5
Eppig [39]	normal	6.0 ± 5.0	6.0 ± 5.0	-	-	0.2 ± 0.2	0.4 ± 0.4
	KCN	34.0 ± 30.0	37.0 ± 32.0	-	-	3.8 ± 4.0	2.0 ± 1.7
Saad * [35]	normal	5.4 ± 4.9	6.0 ± 5.0	0.3 ± 0.3	0.4 ± 0.3	-	0.3 ± 0.3
	KCN	33.9 ± 37.0	35.7 ± 34.5	4.1 ± 2.9	2.4 ± 2.9	-	2.1 ± 2.3
Current Project ^†^	Cluster 1	8.0 ± 6.3	8.1 ± 6.4	0.3 ± 0.3	0.3 ± 0.3	0.3 ± 0.2	0.3 ± 0.3
	Cluster 2	11.3 ± 10.5	11.9 ± 10.8	0.7 ± 0.7	0.8 ± 0.8	0.6 ± 0.5	1.0 ± 1.1
	Cluster 3	41.1 ± 53.6	58.3 ± 92.5	2.5 ± 3.3	3.1 ± 3.8	2.3 ± 2.6	3.4 ± 4.9

* Used Orbscan IIz; all other studies used Pentacam. ^†^ Excluding 571 cases with quality error > 0 in either eye. See Table 3 for full sample results.

## Data Availability

Restrictions apply to the availability of these data. Data was obtained from the Shahroud Eye Cohort Study.

## Supplementary Materials

The following are available online at https://www.mdpi.com/article/10.3390/healthcare9121738/s1, Table S1: Device-generated quality and normality indicators extracted from each CSV file and their recoding into fewer categories, Table S2: Combining the recoded quality and normality indicators into 6 bilateral categories, and Table S3: The mean ± standard deviation (central 95% range) of the descriptive statistics of the interocular elevation difference values (µm) in the central 2.0 mm–6.0 mm zones of the cornea within each individual (*n* = 4613).
