# Supplementary material for: Interocular Symmetry Analysis of Corneal Elevation Using the Fellow Eye as the Reference Surface and Machine Learning
Source: Healthcare (Basel). 2021 Dec 16;9(12):1738. doi: 10.3390/healthcare9121738 (PMC8702115; doi:10.3390/healthcare9121738)
Supplement: Supplementary file 1 [file healthcare-09-01738-s001.zip › healthcare-1512249-supplementary.pdf]

healthcare

MDPI

Supplementary

Table S1. Device-generated quality and normality indicators extracted from each CSV file and their recoding into fewer categories.

Variable	Indicative of	Unit/Categories	New Code
QS Error	Error during imaging	0	OK
		1, 2	Error
KKS	KCN grade or other abnormality	0	Normal
		1, 1–2, 2	KCN1–2
		2–3, 3, 3–4, 4	KCN3–4
		Possible, Abnormal, Cataract Surgery?	Abnormal

QS: quality specification; KKS: keratoconus score; KCN: keratoconus.

Table S2. Combining the recoded quality and normality indicators into 6 bilateral categories.

KKS	QS Error	Bilateral Category
Normal (bilateral)	OK (bilateral)	Bilateral-normal/QS-OK
KCN1–2 (at least 1 eye)		KCN-1-2/QS-OK
KCN3–4 (at least 1 eye)		KCN-3-4/QS-OK
Abnormal (at least 1 eye)		KS-abnormal/QS-OK
Abnormal (at least 1 eye)	Error (at least 1 eye)	Bilateral-normal/QS-error
Abnormal (at least 1 eye)		KS-abnormal/QS-error

KKS: keratoconus score; QS: quality specification; KCN: keratoconus. Note: If one eye was KCN and the fellow eye Abnormal, it was placed in the KCN category.

Table S3. The mean ±standard deviation (central 95% range) of the descriptive statistics of the interocular elevation difference values (μm) in the central 2.0 mm – 6.0 mm zones of the cornea within each individual (n=4613).

Statistic	2.0 mm	3.0 mm	4.0 mm	5.0 mm	6.0 mm
Data points	317	709	1257	1961	2821
Skew	0.0 ± 0.5 (1.9)	0.0 ± 0.6 (2.2)	0.0 ± 0.9 (3.4)	0.1 ± 1.6 (6.4)	0.3 ± 2.5 (9.7)
Abs-Skew	0.4 ± 0.3 (1.2)	0.5 ± 0.3 (1.3)	0.6 ± 0.7 (2.6)	0.7 ± 1.5 (5.8)	1.0 ± 2.3 (9.0)
Kurtosis	−0.1 ± 0.8 (3.2)	0.0 ± 0.7 (2.8)	0.6 ± 10.0 (39.3)	3.0 ± 50.3 (197.3)	7.2 ± 77.7 (304.8)
Mean	0.0 ± 2.1 (8.3)	0.0 ± 2.5 (9.7)	0.0 ± 3.1 (12.0)	0.0 ± 3.9 (15.3)	0.2 ± 5.4 (21.3)
SD	1.3 ± 2.0 (7.9)	2.0 ± 3.1 (12.3)	2.9 ± 4.5 (17.8)	4.4 ± 6.9 (27.0)	7.3 ± 13.3 (52.1)
Abs-Mean	0.5 ± 2.1 (8.1)	0.8 ± 2.3 (9.1)	1.3 ± 2.8 (11.0)	1.8 ± 3.5 (13.6)	2.6 ± 4.8 (18.7)
Mean-Abs	1.03 ± 2.4 (9.4)	1.7 ± 3.1 (12.2)	2.5 ± 4.1 (16.1)	3.6 ± 5.3 (20.8)	5.0 ± 7.1 (27.7)
Median	0.0 ± 2.0 (7.7)	0.0 ± 2.2 (8.7)	0.0 ± 2.6 (10.3)	−0.1 ± 3.1 (12.2)	−0.1 ± 3.7 (14.3)
Abs-Median	0.3 ± 1.9 (7.6)	0.6 ± 2.1 (8.3)	1.0 ± 2.4 (9.5)	1.3 ± 2.8 (11.1)	1.7 ± 3.2 (12.7)
Minimum	−2.8 ± 3.6 (14.0)	−4.8 ± 6.6 (25.9)	−7.5 ± 10.5 (41.0)	−11.1 ± 15.1 (59.3)	−16.0 ± 20.9 (81.9)
Maximum	2.9 ± 6.1 (24.1)	4.8 ± 9.1 (35.7)	7.8 ± 15.4 (60.3)	13.6 ± 35.5 (139.3)	31.3 ± 92.6 (363.0)
Abs-Maximum	3.7 ± 6.5 (25.4)	6.3 ± 9.8 (38.5)	10.2 ± 16.5 (64.5)	17.4 ± 36.4 (142.5)	36.8 ± 92.4 (362.3)
Range	5.7 ± 8.6 (33.7)	9.6 ± 14.3 (56.0)	15.3 ± 23.0 (90.1)	24.7 ± 43.3 (169.7)	47.3 ± 99.3 (389.1)
Central 95% Range	5.0 ± 7.9 (31.0)	7.8 ± 12.3 (48.4)	11.6 ± 17.8 (69.8)	17.1 ± 27.0 (105.7)	28.5 ± 52.1 (204.1)
Negative Volume	−1.6 ± 2.4 (9.6)	−6.0 ± 9.5 (37.3)	−16.1 ± 25.7 (100.7)	−35.4 ± 55.4 (217.3)	−68.4 ± 104.1 (407.9)
Positive Volume	1.7 ± 6.8 (26.5)	6.0 ± 17.5 (68.4)	15.7 ± 37.5 (147.2)	34.6 ± 72.1 (282.8)	73.6 ± 143.3 (561.8)
Total Volume	3.3 ± 7.6 (29.8)	12.1 ± 22.0 (86.2)	31.8 ± 51.5 (201.8)	69.9 ± 103.8 (407.0)	142.0 ± 199.5 (782.1)
Volume Difference	0.1 ± 6.7 (26.4)	0.0 ± 17.5 (68.8)	−0.4 ± 38.6 (151.2)	−0.8 ± 76.0 (297.8)	5.2 ± 151.4 (593.7)

Abs: absolute value; SD: standard deviation; Mean-Abs: Average of absolute differences; Abs-Maximum: the larger of the maximum and absolute minimum.
